# Supplementary material for: The Role of Macrophage Inhibitory Factor in TAA-Induced Liver Fibrosis in Mice: Modulatory Effects of Betaine
Source: Biomedicines. 2024 Jun 17;12(6):1337. doi: 10.3390/biomedicines12061337 (PMC11201963; doi:10.3390/biomedicines12061337)
Supplement: Supplementary file 1 [file biomedicines-12-01337-s001.zip › biomedicines-3023724-supplementary.pdf]

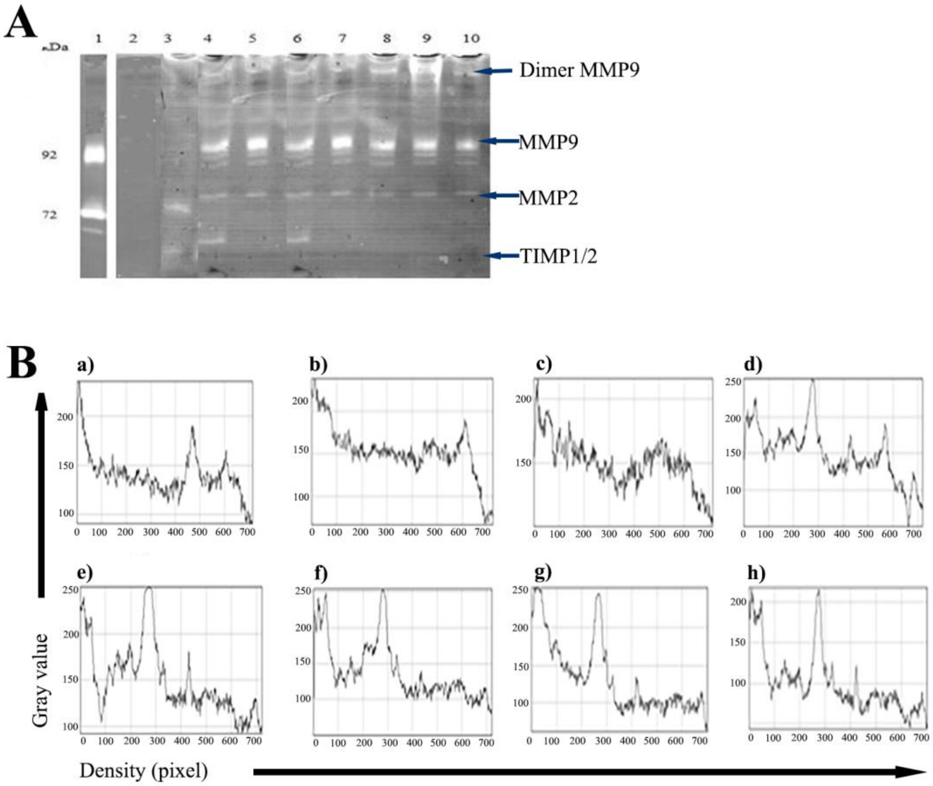

Figure S1. Gelatin zymography of MMPs of tissue homogenates of thioacetamide induced liver fibrosis in mice. (A) Gelatin zymogram. Liver tissue homogenates (LTH) were processed as described in Materials and Methods Section and analyzed by gelatin zymography. LTH samples of all groups shown above each lane: 1. Standard; 2. Standard inhibited with EDTA; 3. Control; 4. and 6.TAA; 5. and 8. TAA+Bet; 7. and 9. MIF$^{-/-}$+TAA; 10. MIF$^{-/-}$+ TAA + Bet. (B) Densitograms of gelatin zymography of MMPs in tissue homogenates of TAA induced liver fibrosis in mice. a) C, b) Bet, c) MIF$^{-/-}$, d) MIF$^{-/-}$; e) TAA; f) TAA+Bet; g) MIF$^{-/-}$+TAA; h) MIF$^{-/-}$+ TAA + Bet. For abbreviations see Figure 1.
